# Deep learning enables automated detection of dinosaur footprints with high accuracy

**DOI:** 10.1038/s41598-026-56031-5

**Published:** 2026-06-04

**Authors:** Yeoncheol Ha, Seung-Sep Kim

**Affiliations:** 1 Department of Earth, Environmental and Space Sciences, Chungnam National University, 99 Daehak-ro, Yuseong-gu, Daejeon, 34134 Republic of Korea; 2 Department of Geological Sciences, Chungnam National University, 99 Daehak-ro, Yuseong-gu, Daejeon, 34134 Republic of Korea

**Keywords:** Dinosaur footprints, Deep learning, Object detection, YOLOv8, Paleontology, Ichnology, Ecology, Ecology, Evolution

## Abstract

Dinosaur footprints provide crucial paleoecological information about locomotion, behavior, and tracksite distributions. Traditional tracksite surveys rely on time-consuming manual identification by expert researchers, with results influenced by preservation quality and subjective interpretation. Here, we present an automated detection system for dinosaur footprints using You Only Look Once version 8 (YOLOv8), an effective object detection neural network. We trained the model on 49,242 images from the AI-Hub dataset, comprising theropod, ornithopod, and sauropod footprints from Korean tracksites. The final model achieved a mean average precision (mAP50) of 0.949 and mAP50-95 of 0.660. To address resolution-dependent detection challenges, we developed a multi-scale detection approach combining predictions across eight different image resolutions. Testing on previously unseen tracksites demonstrated successful detection of footprints from both Korea and Brazil, including the ichnospecies *Farlowichnus rapidus*, indicating strong cross-regional generalization. Our results show that detection accuracy depends on illumination conditions, preservation quality, and the presence of outline markings. This deep learning approach offers an efficient alternative to manual tracksite exploration, enabling rapid spatial distribution mapping of extensive dinosaur tracksites while providing objective, reproducible detection results.

## Introduction

Dinosaur footprints are ethological structures formed when the autopodia of dinosaurs modify a substrate^1^. While body fossils provide information about morphology and size, footprints offer unique paleoecological insights including pace, stride, gait, and locomotion speed^2–4^. The abundance and spatial distribution of footprints at a tracksite are essential for reconstructing dinosaur behavior and paleoenvironments^5^.

Traditionally, tracksite surveys require researchers to visually identify and manually mark footprint outlines for mapping, a process that relies entirely on individual expertise^4^. Recent advances in three-dimensional documentation using Light Detection and Ranging (LiDAR) and photogrammetry have significantly improved analytical accuracy^6–9^. However, regardless of the documentation method employed, factors such as preservation quality and atypical morphologies continue to influence researcher judgment^4^, making traditional survey approaches inherently time-consuming and subjective.

Paleontology has increasingly adopted computational techniques, from basic statistical methods to advanced artificial intelligence (AI) applications^10^. Representative examples of fundamental analytical approaches include the Paleobiology Database (PBDB)^11^, geometric morphometrics^12^, regression analysis^13^, and finite element analysis (FEA)^14^. More recently, AI methods such as convolutional neural networks (CNNs)^15^ have proven particularly effective for analyzing the visual characteristics of dinosaur footprints. For example, Visual Geometry Group-16 (VGG16) based networks have been employed to discriminate between theropod and ornithischian footprints^10^, and Xception networks have been used to classify ornithopod footprint outlines^16^.

With the exception of recent unsupervised approaches^17^, these classification-based methods typically rely on pre-defined, human-produced outlines, which can inadvertently introduce subjective bias at the data-preparation stage. Furthermore, such methods are generally limited to analyzing isolated specimens and are therefore insufficient for tracksite-scale images in which multiple footprints must be detected simultaneously. To address these challenges, the present study prioritizes objective detection of multiple footprints within single images over morphological discrimination.

Here, we address this limitation by developing an automated detection system using You Only Look Once version 8 (YOLOv8)^18^, an efficient one-stage object detector. We trained the model on over 49,000 images of dinosaur footprints from Korean tracksites and validated its performance on previously unseen localities, including sites in Brazil. Our approach enables rapid, objective detection of multiple footprints within single images, offering an efficient alternative to manual tracksite exploration.

## Results

### Model training performance

YOLOv8 is offered in five sizes, ranging from a lightweight “nano” version designed for fast on-device use to an extra-large version designed for the highest possible accuracy. The nano variant (YOLOv8n) uses approximately 3.2 million internal parameters, whereas the extra-large variant (YOLOv8x) uses approximately 68.2 million — about twenty times larger — giving it substantially greater capacity to learn subtle visual features. Larger variants generally achieve higher detection accuracy than smaller ones, at the cost of slower processing. Because dinosaur footprints often present subtle morphological relief and complex surface textures, and because our analysis is performed retrospectively on photographs rather than in real time during fieldwork, we selected YOLOv8x to prioritize accuracy over speed^18^.

**Fig. 1:**
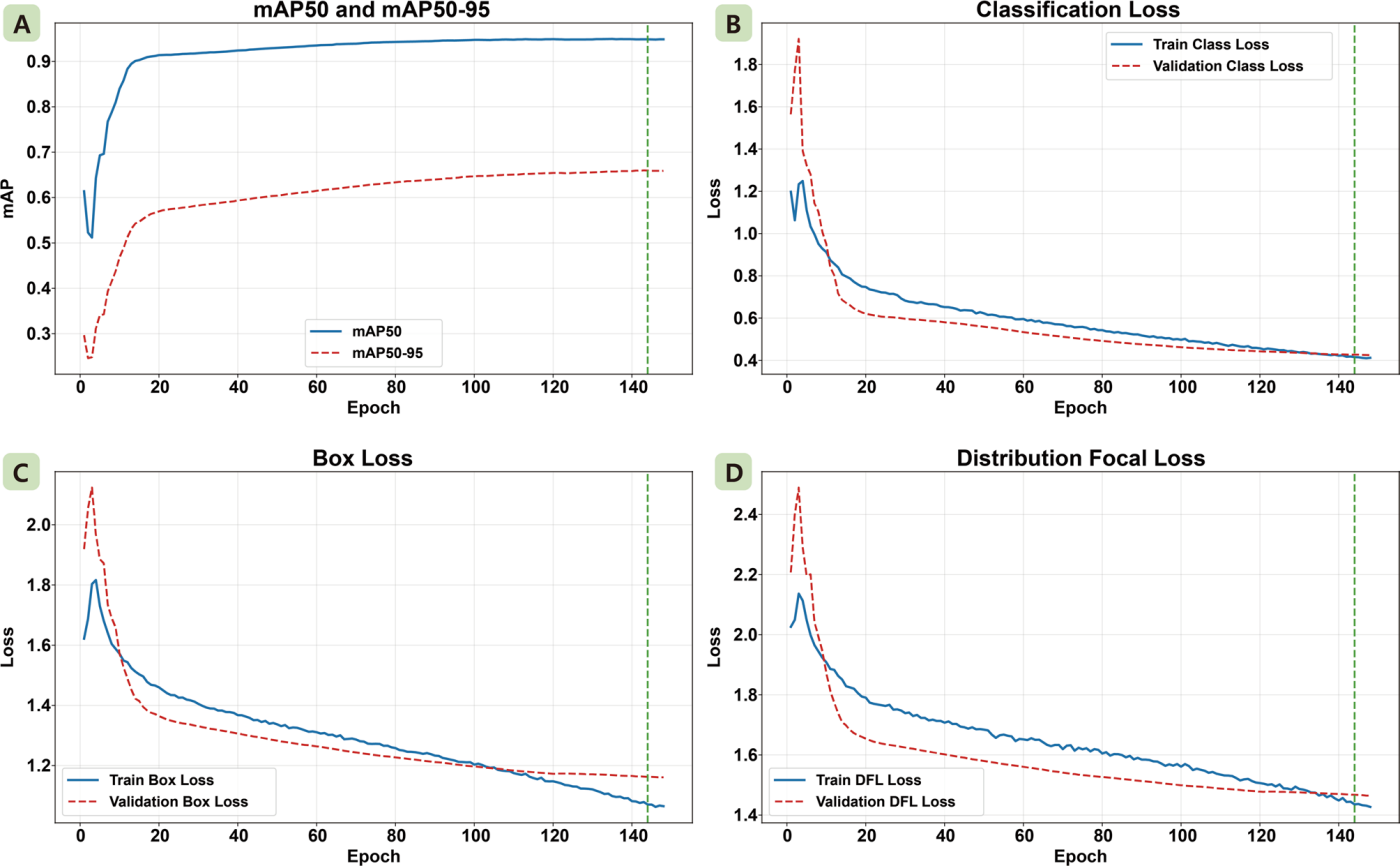
Training performance of the YOLOv8 model for dinosaur footprint detection. (**A**) Mean average precision at IoU (Intersection over Union) threshold 0.50 (mAP50, solid blue line) and IoU thresholds 0.50–0.95 (mAP50-95, dashed red line) on the validation dataset across training epochs. The model achieved final values of mAP50 = 0.949 and mAP50-95 = 0.660. (**B**) Classification loss for training (solid blue) and validation (dashed red) datasets, showing convergence of both curves. (**C**) Box loss (bounding box regression) for training and validation datasets. (**D**) Distribution Focal Loss (DFL) for training and validation datasets. The green dashed vertical line indicates epoch 144, where training was terminated by early stopping after five consecutive epochs without improvement in the fitness metric. All loss functions show consistent decrease without signs of overfitting, as validation losses closely track training losses throughout the training process.

The YOLOv8x model was trained for 149 epochs out of the maximum 300 epochs specified, where one epoch is a complete pass of the entire training dataset through the neural network. Training was terminated by the early stopping function after five consecutive epochs without improvement in the fitness value. The final model, selected from epoch 144, achieved a mean average precision (mAP) at IoU (Intersection over Union) 0.50 (mAP50) of 0.949 and mAP50-95 of 0.660 (Fig. 1).

### Multi-scale detection approach

The training image size within the YOLO framework was set to 640 × 640 pixels. Most images in the dataset contained one or two dinosaur footprints occupying the majority of the image area. When images containing multiple footprints are resized to 640 × 640 pixels for prediction, the features of smaller footprints become diminished in the extracted feature maps, leading to misdetection. This issue is highly dependent on the size of footprints relative to the image dimensions.

**Fig. 2:**
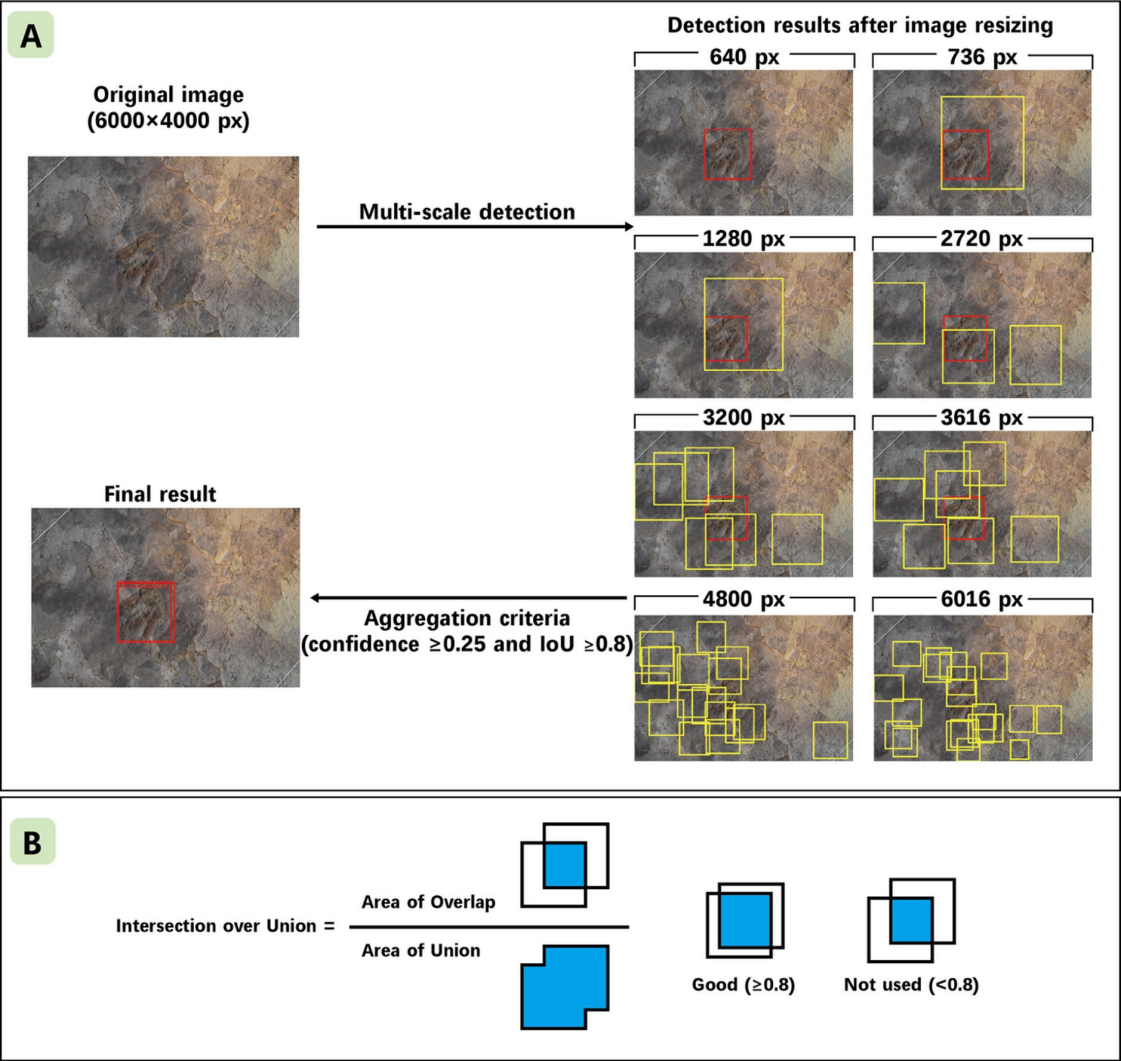
Multi-scale detection workflow and bounding box aggregation using Intersection over Union (IoU). (**A**) Schematic representation of the multi-scale detection approach. The original image (6000 × 4000 pixels) is resized to eight different resolutions (640 × 640, 736 × 736, 1280 × 1280, 2720 × 2720, 3200 × 3200, 3616 × 3616, 4800 × 4800, and 6016 × 6016 pixels), and detection is performed independently at each scale. Yellow boxes indicate individual detections at each resolution; red boxes indicate detections that were consistently identified across multiple scales. Bounding boxes with confidence ≥ 0.25 are retained from each resolution, and boxes with IoU ≥ 0.8 across scales are aggregated. Only detections appearing at four or more resolutions are included in the final result. (**B**) Definition of Intersection over Union (IoU), calculated as the area of overlap divided by the area of union between two bounding boxes. Detections with IoU ≥ 0.8 were considered matching across scales; detections with IoU < 0.8 were excluded from aggregation.

To address this resolution-dependent limitation, we developed a multi-scale detection approach (Fig. 2A). Detection was performed at eight scales: 640 × 640, 736 × 736, 1280 × 1280, 2720 × 2720, 3200 × 3200, 3616 × 3616, 4800 × 4800, and 6016 × 6016 pixels. These resolutions were chosen to satisfy three criteria. First, each value is a multiple of 32, which is required by the YOLOv8 architecture^18^: the network’s maximum stride means that input dimensions must be divisible by 32. Second, the range was set to span from the native training resolution (640 pixels) up to the original size of the test imagery (6016 pixels, the closest multiple of 32 to the 6000-pixel longer edge of the field photographs), so that footprints occupying widely different fractions of the image area would each fall within the model’s effective receptive field at one or more scales. Third, the intervals were spaced more densely at smaller resolutions, where small or partially preserved footprints are most sensitive to feature loss during downscaling, and more sparsely at larger resolutions, where additional sampling yields diminishing returns. The specific values listed here are illustrative rather than prescriptive: the maximum resolution and the spacing between scales can be adjusted to match the dimensions and footprint densities of other target imagery.

For each resolution, only bounding boxes with confidence values greater than 0.25 (the default YOLOv8 threshold) were retained. The combined bounding boxes from each resolution were compared across all resolutions using IoU, with only those exceeding an IoU threshold of 0.8 considered as matching detections (Fig. 2B). In the final output, only bounding boxes detected at four or more resolutions were retained. Red boxes in Fig. 2A indicate predictions where bounding boxes overlapped more than four times, while yellow boxes show predictions without sufficient overlap.

Notably, higher resolutions do not always yield better detection performance. Excessively high resolutions can hinder detection by introducing noise or computational inefficiencies. Detection results depend not only on resolution but also on the proportion of image area occupied by the footprints. By comparing detection results across multiple resolutions using IoU, both bounding box size and position are evaluated, enabling accurate detection while addressing resolution-dependent misdetection.

### Detection of theropod footprints at Sanbukdong, Gunsan

**Fig. 3:**
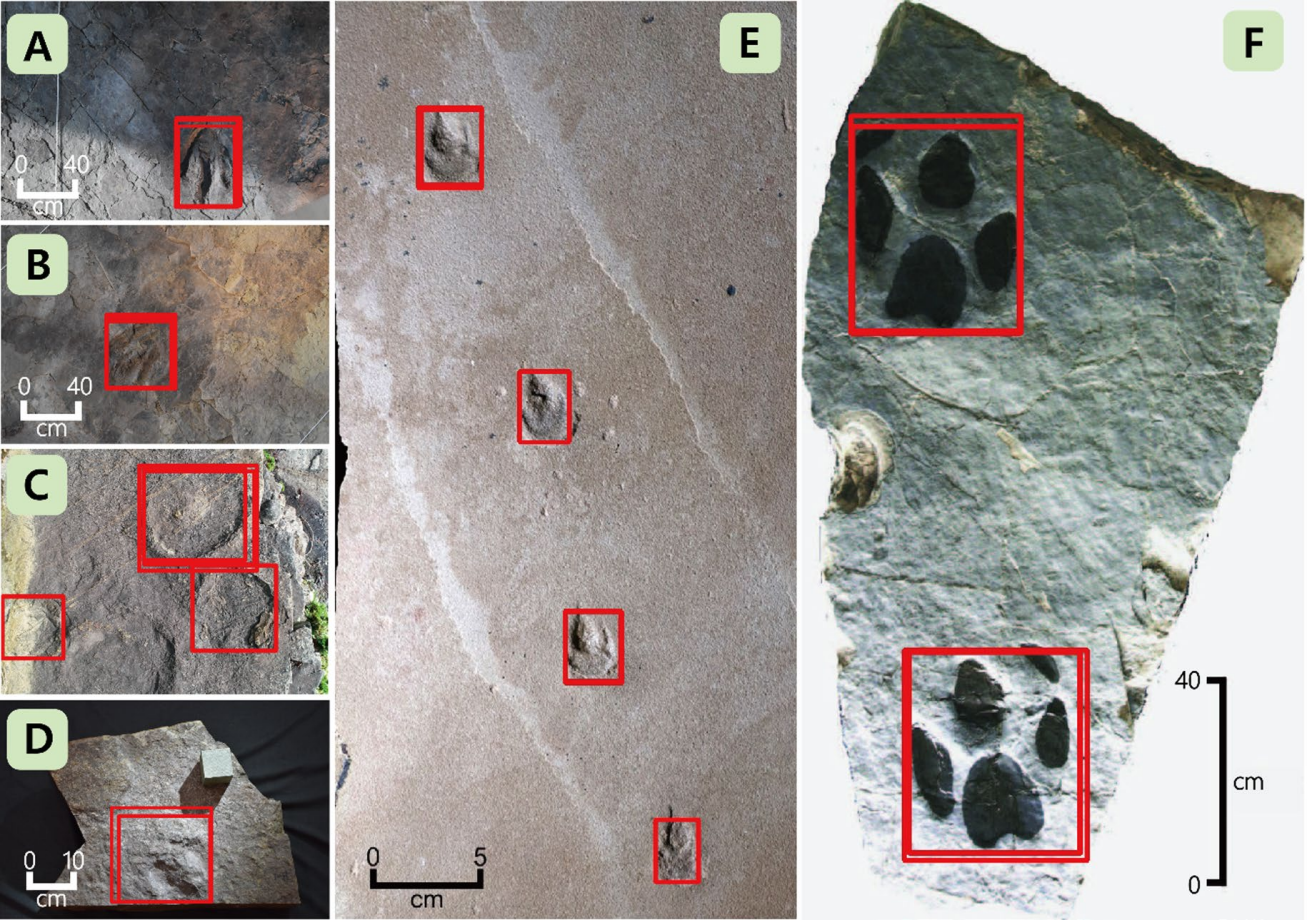
Detection results of the YOLOv8 model on test images from diverse tracksites and preservation conditions. Red boxes show all multi-scale detections that satisfied the aggregation criteria defined in Fig. 2 — that is, bounding boxes appearing at four or more of the eight inference resolutions with pairwise IoU ≥ 0.8. Where multiple overlapping boxes are present, the visible variation in box size reflects the scale tolerance permitted by the IoU threshold rather than independent detections. Reported area percentages give the range of footprint area relative to the full image across these overlapping boxes. (**A**,**B**) Large theropod footprints at Sanbukdong, Gunsan, South Korea (Korea Natural Monument No. 548)^19^, detected from (A) photogrammetric orthomosaic and (B) unprocessed photograph. Footprints occupy 7.13% and 6.89% of the image area, respectively. (**C**) Sauropod pes tracks at Gyeseungsa, Goseong, South Korea (Korea Natural Monument No. 475)^20^. Three of four pes impressions were successfully detected; the undetected track was incompletely preserved. Individual footprints occupy 13.03%, 9.58%, and 5.17% of the image area. (**D**) Theropod footprint cast specimen from Mancheon-ri, Uiseong, South Korea, housed at the Natural History Museum of Chungnam National University (CNUNHM_Fo_030). The well-preserved footprint (11.23% of image area) was detected, while an adjacent poorly preserved impression was not detected. (**E**) *Farlowichnus rapidus* (LPP-IC-0200)^21^ from the Early Cretaceous Botucatu Formation at the Ouro ichnosite, São Paulo, Brazil. All four footprints were successfully detected despite the model being trained exclusively on Late Cretaceous Korean tracksites. Individual footprints occupy 0.84–1.09% of the image area. (**F**) *Hadrosauropodus kyoungsookimi* (NHCG 10194)^22^ trackway from Duho-ri, Goseong, South Korea. Ornithopod pes impressions (8.02–8.03% of image area) were detected; manus impressions were not detected as they were not included in the training dataset.

Various ichnofossils including theropod, ornithopod, and pterosaur tracks have been reported at the Sanbukdong locality in Gunsan, South Korea (Korea Natural Monument No. 548)^19^. We tested the model on large theropod footprints from this site using both photogrammetric data and unprocessed photographs (Fig. 3A, B). The model successfully detected theropod footprints in both cases. The original image size was 6000 × 4000 pixels, with theropod footprints occupying 7.13% (Fig. 3A) and 6.89% (Fig. 3B) of the image area, respectively.

### Detection of sauropod footprints at Gyeseungsa, Goseong

The tracksite at Geumtae Mountain in Daebeop-ri, Yeonghyeon-myeon, Goseong-gun (Korea Natural Monument No. 475) contains numerous sauropod footprints and associated sedimentary structures^20^. The sauropod trackway includes four pes and three manus impressions^20^. The model successfully detected three of the four sauropod pes tracks (Fig. 3C). The undetected footprint was incompletely preserved and did not appear in complete form in the photograph. The original image size was 4864 × 3648 pixels, with individual sauropod footprints occupying 13.03%, 9.58%, and 5.17% of the image area, respectively.

### Detection of theropod footprints from museum specimens

A theropod footprint excavated from Mancheon-ri, Uiseong, is preserved as a cast specimen at the Natural History Museum of Chungnam National University (CNUNHM_Fo_030). The specimen contains two footprints with convex morphology. One footprint exhibits three toes with pointed claw impressions and a distinct V-shaped heel mark characteristic of theropods. The other footprint is poorly preserved and incomplete. The model successfully detected the well-preserved theropod footprint but failed to detect the poorly preserved specimen (Fig. 3D). The original image size was 6000 × 4000 pixels, with the detected theropod footprint occupying 11.23% of the image area.

### Cross-regional validation: *Farlowichnus rapidus* from Brazil

To test the model’s generalization capability beyond Korean tracksites, we applied it to footprints from the Botucatu Formation at the Ouro ichnosite in São Paulo, Brazil^21^. These small theropod tracks, assigned to *Farlowichnus rapidus* (LPP-IC-0200), are characterized by relatively large and wide digit III (the middle toe) impressions, small blade-like outer digits, and digit II (the inner toe) longer than digit IV (the outer toe)^21^. Despite being trained exclusively on Late Cretaceous footprints from South Korea, the model successfully detected all four *Farlowichnus rapidus* footprints from these Early Cretaceous Brazilian strata (Fig. 3E). The original image size was 712 × 1418 pixels, with individual footprints occupying 1.09%, 0.84%, 1.06%, and 0.90% of the image area, respectively.

### Detection of ornithopod trackways

An ornithopod trackway with manus impressions from Duho-ri, Goseong, South Korea, was originally reported as *Caririchnium*^22^ but has since been reclassified as *Hadrosauropodus* based on morphological characteristics^23^. The model successfully detected the ornithopod pes impressions (NHCG 10194) (Fig. 3F). However, manus tracks were not detected because the training dataset did not include manus impressions. The original image size was 690 × 1263 pixels, with ornithopod pes footprints occupying 8.03% and 8.02% of the image area, respectively.

### Effects of illumination and outline markings on detection

Dinosaur footprints occur as both concave (mold) and convex (cast) forms, and their visibility can be significantly affected by lighting conditions. During tracksite mapping, outline marking is often employed as an auxiliary visualization method. The training dataset included footprints with and without outline markings.

**Fig. 4:**
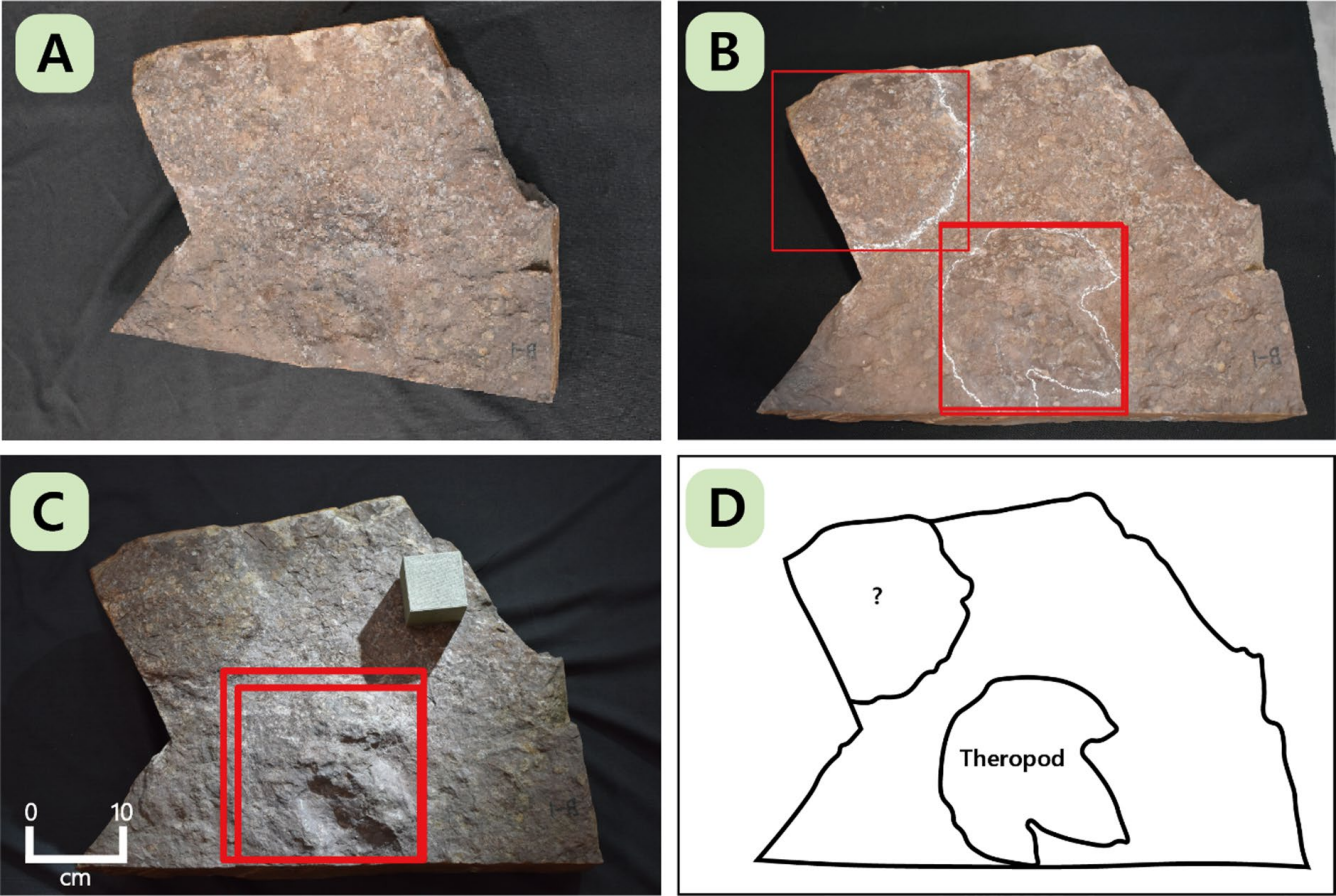
Effects of illumination angle and outline markings on footprint detection performance. (**A**) Vertical illumination without outline markings. Overhead lighting eliminates shadows within the footprint depressions, reducing surface contrast and resulting in no detections. (**B**) Vertical illumination with outline markings. White painted outlines provide sufficient visual distinction for successful detection of both footprints (red boxes), despite the absence of shadow-induced contrast. (**C**) Diagonal illumination without outline markings. Oblique lighting creates shadows that enhance the three-dimensional appearance of footprints; only the well-preserved theropod footprint with distinct morphology was detected, while the poorly preserved impression in the upper portion of the specimen remained undetected. (**D**) Interpretive sketch of the specimen showing footprint locations. The well-preserved tridactyl theropod footprint is labeled; the question mark indicates the poorly preserved impression that was not detected by the model. Testing was applied to the cast specimen (CNUNHM_Fo_030) shown in Fig. 3D.

We examined how illumination angle and outline markings affect detection performance (Fig. 4). When light was directed vertically onto footprints without outline markings, shadows on the footprint surface were eliminated, resulting in failed detection (Fig. 4A). However, when footprints with outline markings were illuminated vertically, all footprints were successfully detected (Fig. 4B). Under diagonal illumination without outline markings, well-preserved theropod footprints were successfully detected, but poorly preserved footprints remained undetected (Fig. 4C). These results demonstrate that detection performance is influenced by the interplay of illumination conditions, preservation quality, and the presence of outline markings.

## Discussion

This study demonstrates that deep learning object detection can effectively automate the identification of dinosaur footprints across diverse tracksites and preservation conditions. The YOLOv8 model achieved high detection accuracy (mAP50 = 0.949, mAP50-95 = 0.660) and successfully identified footprints from theropod, ornithopod, and sauropod trackmakers. The multi-scale detection approach developed here addresses a fundamental challenge in applying fixed-resolution neural networks to variable-sized geological features, enabling reliable detection regardless of footprint size relative to image dimensions.

A notable finding is the model’s ability to generalize beyond its training data. Although trained exclusively on Late Cretaceous footprints from the Gyeongsang Basin of South Korea, the model successfully detected *Farlowichnus rapidus* from Early Cretaceous strata in Brazil^21^. This cross-temporal and cross-regional generalization suggests that the model learned fundamental morphological features common to dinosaur footprints rather than site-specific characteristics. Such generalization capacity is essential for practical applications, as it indicates the model can be deployed at previously unstudied localities without retraining.

Our results reveal that detection performance is influenced by three interacting factors: illumination conditions, preservation quality, and the presence of outline markings. Vertical illumination eliminates the shadows that define footprint morphology, leading to detection failure in unmarked specimens. However, when outline markings are present, detection succeeds regardless of illumination angle. Diagonal illumination enhances detection of well-preserved footprints by creating shadows that accentuate three-dimensional relief, but poorly preserved impressions with subtle topography may still escape detection. These findings have practical implications for image acquisition: we recommend diagonal illumination for unmarked footprints, or vertical illumination when outline markings have been applied.

The automated detection approach offers significant advantages over traditional tracksite survey methods. Conventional workflows require researchers to manually explore tracksites, visually identify footprints, mark outlines, and create distribution maps—a process that is time-consuming, labor-intensive, and dependent on individual expertise^4^. Our deep learning approach enables rapid determination of footprint spatial distributions, potentially reducing survey time from days to hours for extensive tracksites. Furthermore, automated detection provides objective, reproducible results that are not subject to inter-observer variability, addressing a longstanding concern in ichnological studies where footprint identification can be influenced by researcher experience and subjective interpretation.

Rather than re-examining tracksites where footprints have already been confidently identified by experts, the approach is best positioned as a tool for automated discovery on large or underexplored surfaces. Many regionally significant exposures — including coastal platforms, recently exposed bedding planes following erosion, and unmapped tracksites identified during regional geological surveys — are too extensive for systematic visual inspection by a small team of specialists. In such settings, the model can serve as a rapid, first-pass detection step that flags candidate footprints for subsequent expert verification, focusing scarce ichnological expertise on the most informative surfaces. This forward-looking application — automated discovery on extensive or previously unstudied tracksites, rather than confirmation of prior identifications — best reflects how we anticipate the method will be used in practice.

Several limitations should be acknowledged. First, the model employs a single label (“footprint”) and cannot discriminate among trackmaker taxa. While this approach maximizes detection sensitivity, it precludes automated taxonomic classification at the ichnospecies or even broad morphotype level. Second, detection performance depends on adequate visual contrast between footprints and surrounding substrate; poorly preserved impressions or those obscured by surface weathering may escape detection. Third, the model was not trained on ornithopod manus impressions, as demonstrated by the failure to detect ornithopod manus tracks in the *Hadrosauropodus* trackway. Fourth, the current implementation requires post-hoc analysis of photographs rather than real-time field detection. Finally, although the test images used here contain extensive non-track regions that function as implicit negative samples, a dedicated quantitative benchmark of false-positive rates on curated negative datasets — for example, photographs of rock surfaces with desiccation cracks, raindrop impressions, or other pseudofossil structures that may superficially resemble footprints — remains a target for future work.

Such future developments could address these limitations through several approaches. As demonstrated by the illumination experiments (Fig. 4), interpretive surface markings such as chalk outlines can themselves provide sufficient contrast for detection. To reduce the influence of such visual artifacts, future approaches could incorporate three-dimensional topographic data, which would compel the model to rely on intrinsic morphology and relief rather than on colour or interpretive markings. Expanding the training dataset to include taxonomically labeled footprints would enable multi-class classification, allowing simultaneous detection and identification. Incorporating manus impressions and a broader range of preservation states would improve detection completeness. Integration with unmanned aerial vehicle (UAV) photogrammetry would enable efficient survey of large or inaccessible tracksites, combining the spatial coverage of aerial imaging with the analytical power of deep learning. Real-time detection implemented on portable devices could transform field survey workflows, allowing researchers to identify potential footprints during initial site reconnaissance.

In conclusion, this study establishes deep learning as a viable tool for automated dinosaur footprint detection. By addressing challenges inherent in manual multi-track detection processes, computer vision-based approaches offer efficient and objective analysis of extensive tracksite datasets. The application of artificial intelligence in ichnology not only streamlines data collection but also opens avenues for new discoveries and quantitative analyses of dinosaur behavior, locomotion, and paleoecology at scales previously impractical with traditional methods.

## Methods

### YOLOv8 architecture

You Only Look Once (YOLO) is a one-stage object detector first introduced in 2015, designed for real-time detection^24^. The YOLO series has evolved through multiple iterations, with each version addressing limitations of previous implementations^25^. YOLOv8, developed by Ultralytics in 2023, supports detection, segmentation, classification, and pose estimation tasks. The framework offers five model variants based on parameter count: nano, small, medium, large, and extra-large. We employed the extra-large model (YOLOv8x) to maximize detection accuracy.

**Fig. 5:**
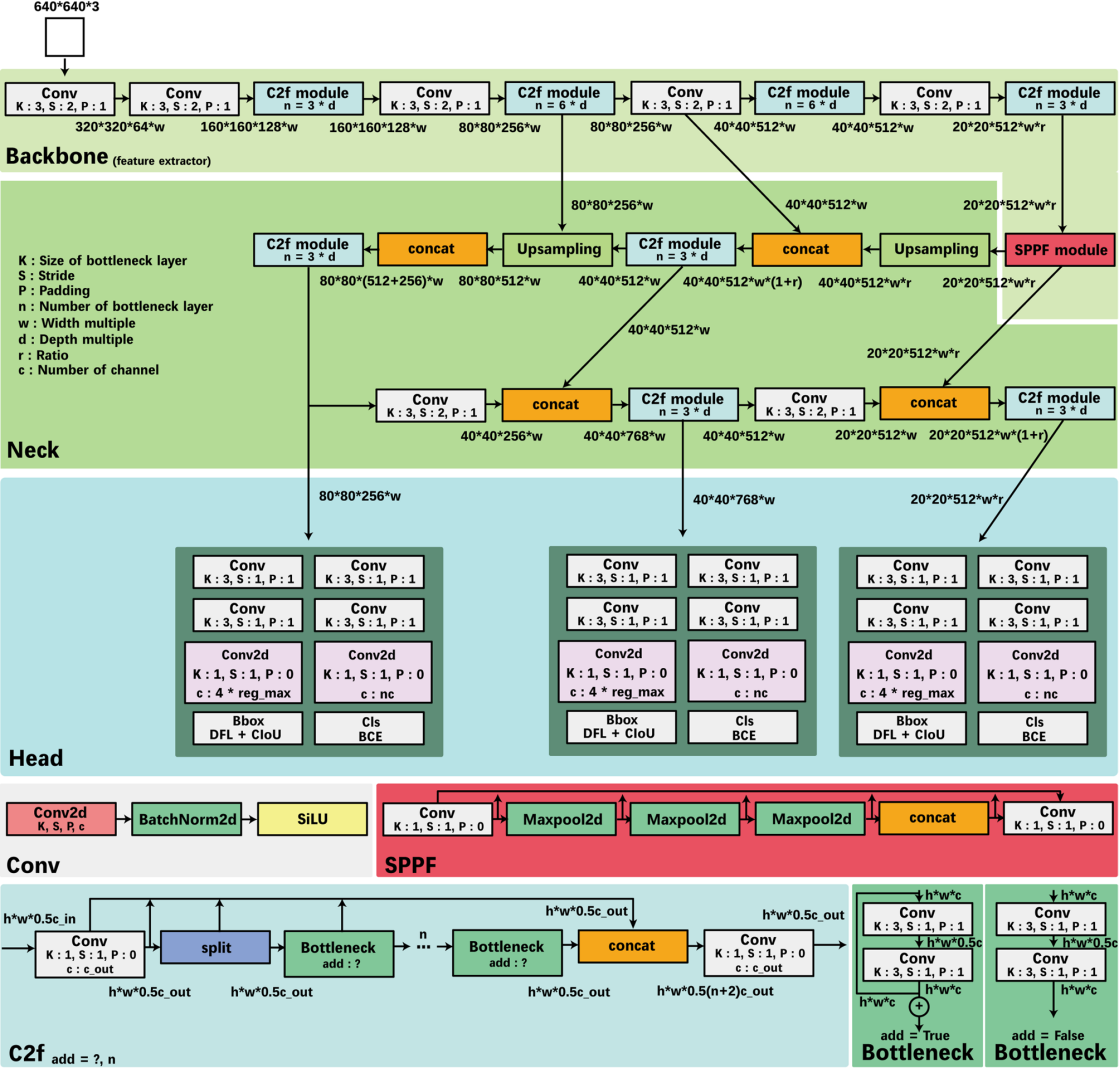
YOLOv8 architecture for dinosaur footprint detection. The network consists of three main components. The **Backbone** extracts features through a series of Conv and C2f modules, progressively reducing spatial dimensions from 640 × 640 to 20 × 20 while increasing channel depth. The **Neck** combines Feature Pyramid Network (FPN) and Path Aggregation Network (PAN) structures: the upper pathway upsamples features from deep to shallow layers to enrich semantic information, while the lower pathway uses strided convolutions to propagate spatial details back to deeper layers; concatenate operations (concat) merge features from different scales. The **Head** employs a decoupled architecture with three detection branches operating at different scales (80 × 80, 40 × 40, and 20 × 20 feature maps). Each branch separates into bounding box regression (Bbox) using Distribution Focal Loss (DFL) and Complete IoU (CIoU) loss, and classification (Cls) using binary cross-entropy (BCE) loss. Bottom panels show module details: **Conv** consists of Conv2d, BatchNorm2d, and SiLU activation; **SPPF** (Spatial Pyramid Pooling-Fast) applies three sequential Maxpool2d operations followed by concatenation; **C2f** splits input features, processes one path through n Bottleneck modules, then concatenates all outputs; **Bottleneck** modules contain two Conv layers with optional shortcut addition (add = True when input and output channels match, add = False otherwise). Notation: K = size of bottleneck layer, S = stride, P = padding, n = number of Bottleneck layers, w = width multiple, d = depth multiple, r = ratio, c = number of channels. Architecture diagram modified after Ultralytics^26^.

The YOLOv8 architecture consists of three components: backbone, neck, and head (Fig. 5). The backbone serves as the feature extractor using a Cross Stage Partial (CSP) network structure^27^. The CSP architecture addresses redundant gradient information in deep networks by splitting feature maps into two parts, processing them through separate paths, and merging them to enhance gradient flow variability, thereby improving computational efficiency and learning capability.

The YOLOv8 backbone implements the CSP structure through C2f (Cross Stage Partial with two convolutions) modules^27^. Each C2f module processes input through a convolutional block, splits the resulting feature map into two parts, passes one part through a series of Bottleneck modules while the other bypasses directly to a concatenation layer, then merges all outputs through a final convolutional block. The number of Bottleneck modules is determined by the depth multiple parameter. Each convolutional module consists of a convolutional layer, batch normalization, and the Sigmoid Linear Unit (SiLU) activation function^28^. The SiLU activation function is characterized by smoothness, non-monotonicity, unboundedness above, and boundedness below, supporting stable learning for datasets with diverse characteristics.

Bottleneck modules consist of two convolutional modules with an optional shortcut connection. When activated, the shortcut connection provides a direct path for gradients, allowing them to bypass convolutional layers and propagate more effectively during backpropagation. This mitigates the vanishing gradient problem that can hinder effective weight updates in deep neural networks. The Spatial Pyramid Pooling-Fast (SPPF) module, consisting of two convolutional modules and three sequential MaxPool2D layers, enhances feature map coverage by combining serial and parallel approaches in spatial pyramid pooling while reducing computational costs compared to standard Spatial Pyramid Pooling^29,30^.

The neck combines multi-scale features using Feature Pyramid Network (FPN)^31^ and Path Aggregation Network (PAN)^32^ structures. While deeper networks capture more feature information for dense predictions, excessive depth can hinder object localization and cause information loss for small objects^33^. FPN addresses this by upsampling from top to bottom, increasing feature information in lower feature maps, while PAN downsamples from bottom to top to enhance upper feature map information^33^. The merged outputs ensure precise predictions across multiple object sizes. Upsampling layers double feature map dimensions without changing channel numbers, enabling effective detection of objects at varying scales.

The YOLOv8 head adopts a decoupled architecture in which classification and detection branches are separated^34^. The head uses an anchor-free approach^35^, predicting object centers and regressing bounding box offsets rather than relying on predefined anchor boxes, enhancing detection efficiency and flexibility. Each detection block contains two branches for classification and bounding box prediction, each consisting of two convolutional modules and a Conv2D layer that generates the respective loss values.

YOLOv8 employs Distribution Focal Loss (DFL)^36^ and Complete Intersection over Union (CIoU) loss^37^ for bounding box regression, combined with binary cross-entropy for classification, improving detection of relatively small objects^25^.

**Fig. 6:**
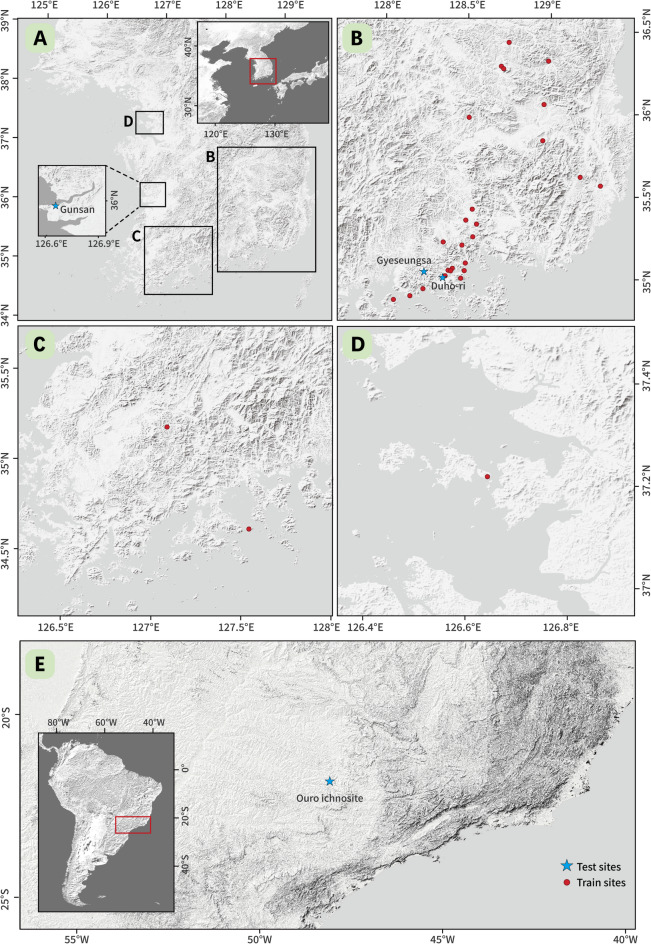
Geographic distribution of dinosaur footprint localities used in this study. (**A**) Overview map of South Korea showing the locations of tracksite regions. Inset at upper right shows the position of the Korean Peninsula in East Asia; inset at lower left shows the Gunsan test locality in detail. Black rectangles indicate areas enlarged in (**B**–**D**). (**B**) Distribution of tracksites in the Gyeongsang Basin, southeastern South Korea, where the majority of training dataset images originated. Test localities Gyeseungsa and Duho-ri are labeled. (**C**) Tracksites in the southwestern coastal region of South Korea. (**D**) Tracksites in the western coastal region near Ansan-si. (**E**) Location of the Ouro ichnosite in São Paulo State, Brazil, used for cross-regional validation. Inset shows the position within South America; the red rectangle indicates the area enlarged in the main panel. Red circles indicate training data localities from the AI-Hub dataset; blue stars indicate test localities not included in training. Shaded relief base maps were generated from Shuttle Radar Topography Mission (SRTM) Global elevation data (NASA, 2013; distributed by OpenTopography, 10.5069/G9445JDF, accessed 26 May 2026) in QGIS version 3.40.14 (10.5281/zenodo.17987821).

### Dataset

We used the dinosaur footprint image dataset provided by AI-Hub (www.aihub.or.kr), established through the Open AI Dataset Project, South Korea. The dataset comprises photographs with polygon annotations of dinosaur footprints from tracksites primarily in the Gyeongsang Basin (Fig. 6). Polygon annotations were converted to bounding boxes for compatibility with the YOLO framework. Original taxonomic annotations (theropod, ornithopod, sauropod) were consolidated into a single label (“footprint”) to maximize detection sensitivity, while structures similar to but distinct from dinosaur footprints were excluded. The final dataset comprised 49,242 images for training and 6,180 images for validation.

The dataset includes three footprint categories. Theropod pes tracks in the dataset are exclusively tridactyl, characterized by impressions longer than wide, tapered digit tips, and V-shaped heel marks. Both tridactyl and didactyl theropod footprints have been reported in Korea^38–42^, but the dataset contains only tridactyl forms. Ornithopod pes tracks are tridactyl with approximately equal length and width, blunt digit terminations, and U-shaped heel marks; poorly preserved specimens may appear nearly circular. Although ornithopod trackways with manus impressions have been documented in Korea^4,22^, manus tracks were not included in the dataset annotations. Sauropod pes tracks are subcircular pentadactyl impressions, though poorly preserved examples may appear circular or semicircular without distinct digit marks.

### Data preprocessing and augmentation

**Fig. 7:**
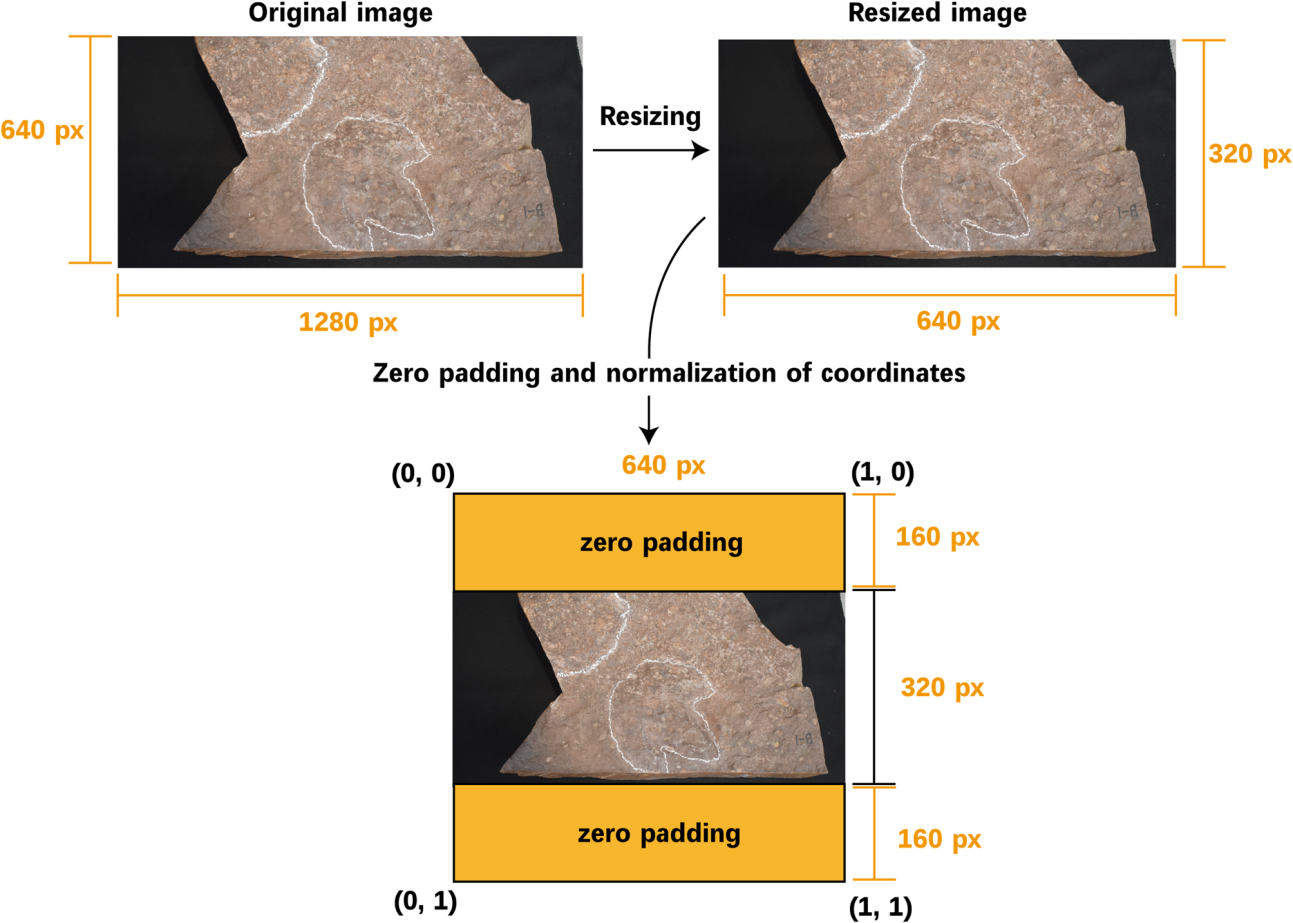
Image preprocessing workflow for rectangular input images in the YOLOv8 framework. The original rectangular image (1280 × 640 pixels) is first resized based on the longer dimension to fit the training size of 640 pixels, producing a 640 × 320 pixel image while maintaining the original aspect ratio. Zero padding is then applied symmetrically to the shorter dimension (160 pixels on top and bottom) to create a square 640 × 640 pixel input. Coordinates are normalized to the range [0, 1] for both axes, with (0, 0) at the top-left corner and (1, 1) at the bottom-right corner. This rectangular training mode (rect mode) preserves footprint morphology by avoiding distortion from forced square resizing, while the zero padding minimizes interference with convolution operations during training.

Most images in the dataset have rectangular aspect ratios. Training in the YOLO framework without preprocessing causes distortion due to forced resizing into square dimensions. To address this, we employed the rectangular training mode (rect mode) of YOLOv8, which resizes images to squares based on the longer dimension and fills the shorter dimension with zero padding. This approach minimizes the impact of empty space on convolution operations during training (Fig. 7).

Data augmentation was implemented using the AutoAugment technique^43^, which employs a Recurrent Neural Network controller to identify optimal augmentation strategies with their respective magnitudes and probabilities. The following augmentation options were applied: mosaic augmentation combining four images into single composites to enhance generalization across diverse scenes; HSV modifications adjusting hue, saturation, and brightness to improve robustness under varying lighting conditions; translation shifting images vertically or horizontally to enhance detection of partial objects; scaling to improve detection at various distances; and horizontal flipping to increase object diversity.

### Training configuration

The model was configured with an input image size of 640 × 640 pixels. We employed transfer learning using COCO-pretrained weights, with a batch size of 16 and maximum of 300 epochs. To prevent overfitting, early stopping was implemented to terminate training after five consecutive epochs without improvement in the fitness metric. The fitness value was calculated as the weighted sum of mAP50 (10%) and mAP50-95 (90%):

$$ \tt Fitness = 0.1 \times mAP50 + 0.9 \times mAP50\mathrm{-}95$$

Training was performed using the Ultralytics YOLOv8 framework (https://github.com/ultralytics/ultralytics).

### Multi-scale detection

To address resolution-dependent detection limitations, we implemented a multi-scale detection approach in which inference was performed at eight image resolutions (640 × 640, 736 × 736, 1280 × 1280, 2720 × 2720, 3200 × 3200, 3616 × 3616, 4800 × 4800, and 6016 × 6016 pixels). Detections from all resolutions were aggregated using Intersection over Union (IoU) thresholds, retaining only consistent detections appearing across multiple scales. The detailed workflow and threshold parameters are presented in Results (Fig. 2).

### Evaluation metrics

Model performance was evaluated using mean Average Precision (mAP) at two threshold settings: mAP50, calculated at a single IoU threshold of 0.50, and mAP50-95, averaged across IoU thresholds from 0.50 to 0.95 in increments of 0.05. Precision was calculated as TP/(TP + FP) and recall as TP/(TP + FN), where TP represents true positive detections, FP represents false positive detections, and FN represents false negative (missed) detections.

### Test data

To evaluate model generalization, we tested detection performance on images not included in the training or validation datasets. Test images included theropod footprints from Sanbukdong, Gunsan (Korea Natural Monument No. 548)^19^; sauropod footprints from Gyeseungsa, Goseong (Korea Natural Monument No. 475)^20^; a theropod specimen from Mancheon-ri, Uiseong, housed at the Natural History Museum of Chungnam National University (CNUNHM_Fo_030); *Farlowichnus rapidus* (LPP-IC-0200) from the Botucatu Formation, Brazil^21^; and *Hadrosauropodus kyoungsookimi* (NHCG 10194) from Duho-ri, Goseong^22^. These test images are full-scene field photographs of complex outcrop surfaces rather than tightly cropped views of individual footprints. Each image therefore includes substantial regions of unmarked rock surface, fractures, and non-track sedimentary structures alongside the annotated footprints. These unannotated regions served as implicit negative samples during evaluation, allowing assessment of whether the model could discriminate genuine footprints from visually similar but non-track features under realistic field conditions. Additionally, we examined the effects of illumination angle and outline markings on detection performance using controlled photographs of museum specimens.

## Data Availability

The dinosaur footprint image dataset used in this study is available from AI-Hub (https://aihub.or.kr/aihubdata/data/view.do?dataSetSn=475) under ‘The Open AI Dataset Project (AI-Hub, S. Korea)’. The trained model weights and detection code are available from the corresponding author upon reasonable request.
